# Spike-frequency adaptation inhibits the pairwise spike correlation

**DOI:** 10.3389/fnins.2023.1193930

**Published:** 2023-06-12

**Authors:** Jixuan Wang, Bin Deng, Tianshi Gao, Jiang Wang, Hong Tan

**Affiliations:** ^1^School of Electrical and Information Engineering, Tianjin University, Tianjin, China; ^2^Department of Pathology, The Second Xiangya Hospital, Central South University, Changsha, Hunan, China

**Keywords:** spike frequency adaptation, firing rate, correlation, adaptation conductance, single neuron, pairwise neurons

## Abstract

**Introduction:**

The spike train output correlation with pairwise neurons determines the neural population coding, which depends on the average firing rate of individual neurons. Spike frequency adaptation (SFA), which serves as an essential cellular encoding strategy, modulates the firing rates of individual neurons. However, the mechanism by which the SFA modulates the output correlation of the spike trains remains unclear.

**Methods:**

We introduce a pairwise neuron model that receives correlated inputs to generate spike trains, and the output correlation is qualified using Pearson correlation coefficient. The SFA is modeled using adaptation currents to examine its effect on the output correlation. Moreover, we use dynamic thresholds to explore the effect of SFA on output correlation. Furthermore, a simple phenomenological neuron model with a threshold-linear transfer function is utilized to confirm the effect of SFA on decreasing the output correlation.

**Results:**

The results show that the adaptation currents decreased the output correlation by reducing the firing rate of a single neuron. At the onset of a correlated input, a transient process shows a decrease in interspike intervals (ISIs), resulting in a temporary increase in the correlation. When the adaptation current is sufficiently activated, the correlation reached a steady state, and the ISIs are maintained at higher values. The enhanced adaptation current achieved by increasing the adaptation conductance further reduces the pairwise correlation. While the time and slide windows influence the correlation, they make no difference in the effect of SFA on decreasing the output correlation. Moreover, SFA simulated by dynamic thresholds also decreases the output correlation. Furthermore, the simple phenomenological neuron model with a threshold-linear transfer function confirms the effect of SFA on decreasing the output correlation. The strength of the signal input and the slope of the linear component of the transfer function, the latter of which can be decreased by SFA, could together modulate the strength of the output correlation. Stronger SFA will decrease the slope and hence decrease the output correlation.

**Conclusions:**

The results reveal that the SFA reduces the output correlation with pairwise neurons in the network by reducing the firing rate of individual neurons. This study provides a link between cellular non-linear mechanisms and network coding strategies.

## 1. Introduction

Understanding how the intrinsic properties at the cellular level guide information encoding at the population level is the primary focus of many neural coding studies (Averbeck et al., 2006; Ly et al., 2012). The information carried by neuronal populations is modulated by the correlation between neuronal spike trains (Cohen and Kohn, 2011). Spike trains are widespread in the nervous system and are observed in the thalamus (Alonso et al., 1996), cortex (Zohary et al., 1994), and retina (Shlens et al., 2008). The dominant sources of output correlation are correlation and shared input (de la Rocha et al., 2007; Trong and Rieke, 2008). The given correlated input determines the number of spikes shared between pairwise neurons (Barreiro et al., 2012). For a given network of pairwise neurons, the spike trains of the pairwise neurons are more strongly correlated with higher input correlations or input intensity (Hong et al., 2012). One consequence of output correlation modulation is that it inherits the same variation in the firing rate of individual neurons in a network (de la Rocha et al., 2007). Modulating the average firing rate of individual neurons is key for the network to decode the correlated input and execute information encoding (Litwin-Kumar et al., 2011; Barreiro et al., 2012). However, the mechanism underlying the output correlation modulation by directly altering the firing rate of individual neurons remains unclear. Overcoming this question will help us understand how to control correlation encoding by modulating the firing rate of single neurons.

Spike frequency adaptation (SFA) is a typical neuronal characteristic that encodes information by modulating firing activities (Salaj et al., 2021). The SFA shows active spiking activity following an initial high frequency at the onset of the constant stimulus, but it gradually becomes harder to emit spikes (Benda et al., 2005). This adaptation slows the firing rate (Ha and Cheong, 2017). The dynamic variation in the firing rate causes the spike trains of individual neurons to appear densely distributed initially and then gradually become sparse (Benda and Hennig, 2008). This rich distribution in the spike sequences generated by the SFA changes the shared parts of the sequences continuously (Ramlow and Lindner, 2021). However, it remains unclear how SFA modulates the correlation between pairwise neurons by controlling the shared part. SFA endows single neurons with the ability to vary their firing rates, and this ability varies the spike sequences of pairwise neurons, which further affects output correlation encoding. Therefore, considering the firing rate as the focus factor contributes to the relationship between the SFA and output correlation. This may reveal the mechanism of population encoding at the cellular level.

This study aimed to investigate how SFA, at the cellular level, influences output correlation encoding at the network level. We introduced adaptation currents and dynamic thresholds to explore how the SFA modulates the spike train output correlation. Our results revealed that the SFA decreased the output correlation by reducing the firing rate of single neurons. Our results also provide new insights into possible ways of implementing correlation population encoding by altering cellular mechanisms.

## 2. Materials and methods

### 2.1. Models with different adaptation mechanisms

We are primarily inspired by the *in vitro* experiment that the output correlation is determined by the firing rate (de la Rocha et al., 2007). SFA is a prominent characteristic in decreasing the firing rate, which could be modeled by various physiological adaptation mechanisms, such as *I*_*M*_ (voltage-gated *K*^+^ current; Ermentrout, 1998), *I*_*AHP*_ (*C*^*a*^2^+^ gated *K*^+^ current; Ermentrout, 1998), and *I*_*KNa*_ (*Na*^+^ activated *K*^+^ current; Wang et al., 2003). *I*_*M*_ and *I*_*AHP*_ are inhibitory potassium currents that are activated during APs (Benda and Herz, 2003; Benda et al., 2010). These two kinds of currents shape the information transmission properties on a slow time scale (Ha and Cheong, 2017). *I*_*KNa*_ is mobilized over a longer time scale (Wang et al., 2003). In addition, Benda et al. (2010) reproduced the adaptation by dynamic threshold or adaptation current from the perspective of the physical model. In the following part, three biophysical adaptation mechanisms and two adaptation mechanisms generated by the mathematical model are introduced.

#### 2.1.1. Prescott model

**Figure 1:**
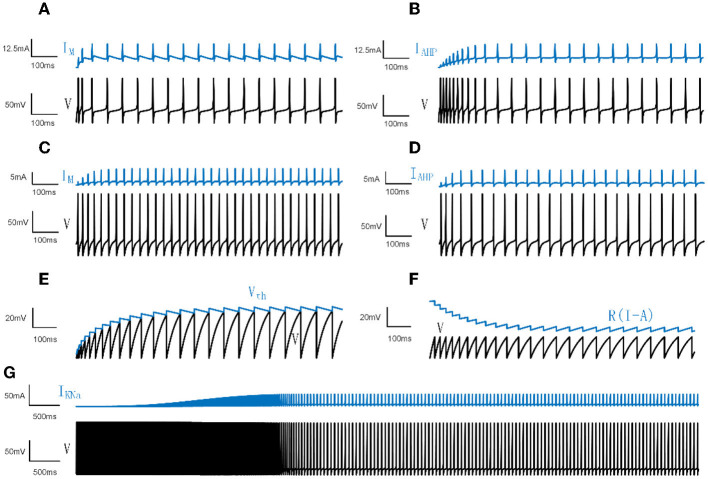
Neural spiking activities and adaptation currents/thresholds for different models. **(A)** The patterns of the membrane potential *V* and M current in the Prescott model. **(B)** The patterns of *V* and AHP current in the Prescott model. **(C)** The patterns of *V* and the M type current in the Ermentrout model. **(D)** The patterns of *V* and the AHP type current in the Ermentrout model. **(E)** The patterns of dynamic threshold *V*_*th*_ and *V* in the LIFDT model. **(F)** The patterns of adaptation current *R*(*I*−*A*) and *V* in the LIFAC model. **(G)** The patterns of *V* and *K*_*Na*_ type current in the Wang model.

The Prescott model is extended by an adaptation current, which is a modified Morris–Lecar model (Morris and Lecar, 1981; Prescott and Sejnowski, 2008). The membrane potential *V* includes inward sodium current, outward potassium current, and passive leak current. The dynamics of membrane potential *V*, the kinetics of the *K*^+^ gating variable *w*, and adaptation variable *z* are described as follows:


(1) $$\begin{array}{l} \begin{array}{lll} C_{m}\frac{dV}{dt} & = & I_{ext}-{\bar{g}}_{Na}m_{\infty }(V)(V-E_{Na}) \end{array}\\ \;\begin{array}{l} -{\bar{g}}_{K}w(V-E_{Na})-{\bar{g}}_{L}(V-E_{L})-I_{adapt} \end{array} \end{array}$$

(2) $$\begin{array}{l} \frac{dw}{dt}=\varphi_{w}\frac{w_{\infty }\left(V\right)-w}{\tau_{w}\left(V\right)} \end{array}$$

(3) $$\begin{array}{l} \frac{dz}{dt}=\frac{z_{\infty }\left(V\right)-z}{\tau_{z}} \end{array}$$

where $C_{m}\text{=}\text{2}\mu \text{F}/\text{c}{\text{m}}^{\text{2}}$ is the membrane conductance. *I*_*ext*_ is the external DC input. *I*_*adapt*_ represents *I*_*M*_ or *I*_*AHP*_. ϕ_*w*_ = 0.15. As manifested in Figures 1A, B, the membrane potential first demonstrates a rapid fluctuation and then presents a continuous steady oscillation. The adaptation current reads:


(4) $$\begin{array}{l} I_{adapt}={\bar{g}}_{adapt}z\left(V-E_{K}\right) \end{array}$$

The maximum conductances are ${\bar{g}}_{Na}\text{=20 mS/c}{\text{m}}^{\text{2}}$, ${\bar{g}}_{K}\text{=20 mS/c}{\text{m}}^{\text{2}}$, and ${\bar{g}}_{L}\text{=2mS/c}{\text{m}}^{\text{2}}$. The reversal potentials are *E*_*Na*_ = 50mV, *E*_*K*_ = −100mV, *E*_*L*_ = −70mV. All parameter settings are based on Prescott and Sejnowski (2008). ${\bar{g}}_{adapt}$ is the adaptation conductance, which is set to different values for different experiments. The time function and steady-state activation are given by:


(5) $$\begin{array}{l} m_{\infty }\left(V\right)=0.5\left[1+\tanh\left(\frac{V-\beta_{m}}{\gamma_{m}}\right)\right] \end{array}$$

(6) $$\begin{array}{l} w_{\infty }\left(V\right)=0.5\left[1+\tanh\left(\frac{V-\beta_{w}}{\gamma_{w}}\right)\right] \end{array}$$

(7) $$\begin{array}{l} \tau_{w}\left(V\right)=1/\cosh\left(\frac{V-\beta_{m}}{2\gamma_{m}}\right) \end{array}$$

(8) $$\begin{array}{l} z_{\infty }\left(V\right)=1/\left[1+\exp\left(\frac{\beta_{z}-V}{\gamma_{z}}\right)\right] \end{array}$$

where β_*m*_ = −1.2mV, γ_*m*_ = 18mV, β_*w*_ = 0mV, γ_*w*_ = 10mV. For the M type current, the parameter settings are ${\bar{g}}_{adapt}={\bar{g}}_{M}\text{=}0.5\text{mS/c}{\text{m}}^{\text{2}},\;\beta_{z}=\text{-}35\text{mV},\;\gamma_{z}=4\text{mV},\;\tau_{z}=100\text{ms}$, which makes *I*_*M*_ activate before action potentials. For the AHP type current, ${\bar{g}}_{adapt}={\bar{g}}_{AHP}\text{=}5\text{mS/c}{\text{m}}^{\text{2}},\;\beta_{z}=0\text{mV},\;\gamma_{z}=4\text{mV},\;\tau_{z}=100\text{ms}$, which enables *I*_*AHP*_ activate during action potentials.

#### 2.1.2. Ermentrout model

The Ermentrout model is a single-compartment model, which is a conductance-based version modified by Traub–Miles model (Shlens et al., 2008). The membrane potential consists of fast *Na*^+^ current *I*_*Na*_, delayed rectifier *K*^+^current *I*_*K*_, voltage-gated *Ca*^2+^ current *I*_*Ca*_, leak current *I*_*L*_, M type current *I*_*M*_, and AHP type current *I*_*AHP*_. The membrane potential follows


(9) $$\begin{array}{l} C_{m}\frac{dV}{dt}=I_{ext}-{\bar{g}}_{Na}m^{3}h(V-E_{Na})-{\bar{g}}_{K}n^{4}(V-E_{Na})-{\bar{g}}_{L}(V-E_{L})\\ \;-{\bar{g}}_{Ca}{\left\{1+\exp[-(V+25)/5\;]\right\}}^{-1}(V-E_{Ca})-I_{M}-I_{AHP} \end{array}$$

where the membrane capacitance $C_{m}=\text{1}\mu \text{F}/\text{c}{\text{m}}^{\text{2}}$. Each adaptation ionic current reads


(10) $$\begin{array}{l} I_{M}={\bar{g}}_{M}z\left(V-E_{K}\right) \end{array}$$

(11) $$\begin{array}{l} I_{AHP}={\bar{g}}_{AHP}\left[Ca^{2+}\right]{\left(30+\left[Ca^{2+}\right]\right)}^{-1}\left(V-E_{K}\right) \end{array}$$

where the maximum conductances are ${\bar{g}}_{Na}\text{=100 mS/c}{\text{m}}^{\text{2}}$, ${\bar{g}}_{K}\text{=80 mS/c}{\text{m}}^{\text{2}}$, ${\bar{g}}_{L}\text{=0}\text{.1 mS/c}{\text{m}}^{\text{2}}$, ${\bar{g}}_{Ca}\text{=1 mS/c}{\text{m}}^{\text{2}}$, ${\bar{g}}_{L}\text{=2mS/c}{\text{m}}^{\text{2}}$. The reversal potentials are *E*_*Na*_ = 50mV, *E*_*K*_ = −100mV, *E*_*L*_ = −70mV, and *E*_*Ca*_ = 120mV. The kinetics of *z* for M type current follows


(12) $$\begin{array}{l} \tau_{z}\frac{dz}{dt}=1/\left\{1+\exp\left[-\left(V+20\right)/5\right]\right\}-z \end{array}$$

where τ_*z*_ = 100ms, and the dynamics of intracellular *Ca*^2+^ concentration [*Ca*^2+^] is given by


(13) $$\begin{array}{l} \frac{d\left[Ca^{2+}\right]}{dt}=-0.002I_{Ca}-0.0125\left[Ca^{2+}\right] \end{array}$$

For the model with AHP type current, $g_{M}=0\text{mS/c}{\text{m}}^{\text{2}}$ and, in a similar way, for the model with M type current, $g_{AHP}=0\text{mS/c}{\text{m}}^{\text{2}}$. The membrane potentials and adaptation currents generated in the Ermentrout model are shown in Figures 1C, D.

#### 2.1.3. Wang model

Different from the Ermentrout model, the Wang model focuses on the sodium-activated potassium current *I*_*KNa*_. We use a single-compartment form proposed by Wang et al. (2003), where the calcium-activated potassium current and the dendritic compartment are not adopted. The kinetics of the membrane potential obeys


(14) $$\begin{array}{l} C_{m}\frac{dV}{dt}=I-{\bar{g}}_{Na}\left\{1+4\exp[-(V+58)/12]\right\}\\ \;{\left\{\exp[-0.1(V+33)]\right\}}^{-3}h(V-E_{Na})\\ \;-{\bar{g}}_{K}n^{4}(V-E_{K})-{\bar{g}}_{L}(V-E_{L})\\ \;-{\bar{g}}_{Ca}{\left\{1+\exp[-(V+20)/9]\right\}}^{-2}(V-E_{Ca})\\ \;-I_{adapt} \end{array}$$

*I*_*KNa*_ results in the firing rate slowing down following an initial high frequency as shown in Figure 1G. The sodium-activated potassium adaptation current is given by:


(15) $$\begin{array}{l} I_{adapt}=0.37{\bar{g}}_{KNa}/\left[1+{\left(38.7/\left[Na^{+}\right]\right)}^{3.5}\right]\left(V-E_{K}\right) \end{array}$$

The gating variables *h* and *n* are given by


(16) $$\begin{array}{l} \frac{dh}{dt}=0.28\exp[-(V+50)/10](1-h)-4/\{\exp[-0.1(V+20)]\\ \;+1\}h \end{array}$$

(17) $$\begin{array}{l} \begin{array}{lll} \frac{dn}{dt} & = & -0.04(V+34)/\{\exp[-0.1(V+34)]-1\}(1-n) \end{array}\\ \;\begin{array}{ll} - & 0.5\;\exp[-(V+44)/25]n \end{array} \end{array}$$

The kinetics of concentration [*Ca*^2+^] and [*Na*^+^] describe as


(18) $$\begin{array}{l} \frac{d\left[Ca^{2+}\right]}{dt}=-0.002I_{Ca}-\left[Ca^{2+}\right]/240 \end{array}$$

(19) $$\begin{array}{l} \frac{d[Na^{+}]}{dt}=-0.0003I_{Na}-0.0018\{{\left[Na^{+}\right]}^{3}/({\left[Na^{+}\right]}^{3}+3375)\\ \;-0.13172\} \end{array}$$

#### 2.1.4. Benda model

The leaky integrate-and-fire neuron with adaptation current (LIFAC) and the leaky integrate-and-fire neuron with a dynamic threshold (LIFDT) are modified from leak integrate-and-fire (LIF). The membrane potential with input current *I*(*t*) is given by


(20) $$\begin{array}{l} \tau_{V}\frac{dV}{dt}=-V+R\left(I\left(t\right)-A\right) \end{array}$$

(21) $$\begin{array}{l} \tau_{A}\frac{dA}{dt}=-A \end{array}$$

where τ_*V*_ = 10ms is the time constant and *R* is the input resistance. *A* is the adaptation current. τ_*A*_ = 100ms is the adaptation time constant. When the membrane potential crosses the threshold, *V* is reset to the value of the resting state. *A* increases with $\Delta A\;\text{=}\;\Delta a{\bar{g}}_{A}c$, which represents the adaptation current in the form of the LIF neuron model.

In addition, the dynamic threshold could generate SFA. The model of LIFDT shows


(22) $$\begin{array}{l} \tau_{V}\frac{dV}{dt}=-V+RI\left(t\right) \end{array}$$

(23) $$\begin{array}{l} \tau_{A}\frac{dH}{dt}=-H\text{+}V_{th} \end{array}$$

Different from the mechanism of adaptation current in LIFAC, the adaptation item is produced by a dynamic voltage threshold *H*. When *V* crosses the threshold *H* rather than *V*_*th*_, *V* is reset to 0 and *H*(*t*+1) = *H*(*t*)+Δ*H*. Δ*H* represents the increment of the change in dynamic threshold. The membrane potentials and adaptation currents of LIFDT and LIFAC are depicted in Figures 1E, F.

### 2.2. Correlation calculation

**Figure 2:**
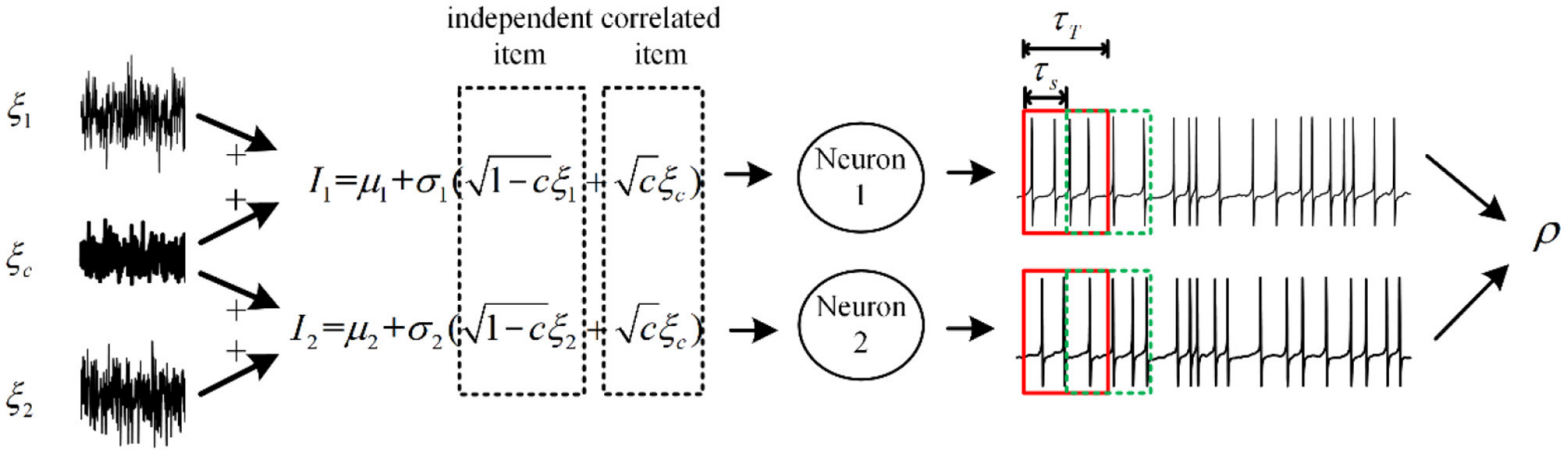
Relationship between correlated input currents and spike train output correlation. The fluctuating inputs follow the Ornstein–Uhlenbeck process with τ = 5*ms*, and some fractions of inputs are shared with each neuron, which is defined by *c*. The input correlation decides the information transition ability from input to output. τ_*T*_ and τ_*s*_ are relevant to the output correlation ρ, which is defined as spike train covariance normalized by variance.

The output correlation is generated by correlated input. We choose a pair of neurons receiving fluctuated correlated input current (Barreiro et al., 2012). As shown in Figure 2, the current has a common input component making the pairwise neuron share afferent input. In addition, the neuron pair has an independent input. The fluctuating input current to neuron *i* (*i* = 1, 2) follows:


(24) $$\begin{array}{l} I_{i}^{k}=\mu_{i}+\sigma_{i}\left(\sqrt{1-c}\xi_{i}^{k}\left(t\right)+\sqrt{c}\xi_{c}^{k}\left(t\right)\right) \end{array}$$

where *k* = 1, 2, ⋯ , *N* (*N* = 1, 000) is the experiment trial and *i* is the neuron index. μ_*i*_ and ${\sigma_{i}}^{2}$ are the average and variance of the input current. *c* (0 ≤ *c* ≤ 1) is the input correlation coefficient. $\xi_{c}^{i}\left(t\right)$ and $\xi_{i}^{k}\left(t\right)$ represent the common and independent Gaussian white noise process of neuron *i* in trial *k*, respectively (Lindner et al., 2005). $\xi_{c}^{i}\left(t\right)$ is the same in different trials, while $\xi_{i}^{k}\left(t\right)$ is randomly generated at each trial. We set appropriate parameters to simulate the variability of $V_{i}^{k}$ (Lampl et al., 1999) and the spike trains generated by $V_{i}^{k}$ (Kohn and Smith, 2005) *in vivo* condition (Alonso et al., 1996). We adopt Pearson's correlation coefficient ρ (Mark Borodovsky, 2008), which is calculated by:


(25) $$\begin{array}{l} \rho \;\text{=}\;\frac{Cov\left(n_{1}^{},n_{2}^{}\right)}{\sqrt{Var\left(n_{1}^{}\right)Var\left(n_{2}^{}\right)}} \end{array}$$

where *Cov* and *Var* represent the covariance and variance. $n_{1}^{}$ and $n_{2}^{}$ are the spike counts computed over the time window τ_*T*_. The common input generates correlation between pairwise neurons. The correlation coefficient ρ depends on two factors: time window τ_*T*_ and slide window τ_*s*_. ρ is a dimensionless quantity, which ranges from 0 (independent) to 1 (entirely correlated), respectively. The input correlation *c* bounds the output correlation ρ. The correlation susceptibility *S* is the slope of ρ to *c*, which is described by:


(26) $$\begin{array}{l} S\;\text{=}\;\rho /c \end{array}$$

where *S* depends only on the input and output correlation. In addition, the coefficient of variation (CV) is adopted to measure the variation in firing rate and the interspike intervals.

The sequence of time intervals between adjacent action potentials of neuronal firing is called the *ISI* sequence. The *ISI* sequence and a histogram of the *ISIs* in the sequence can inform about both the mode and variability of the firing frequency in neuronal activity and are, therefore, useful measures. All simulations are implemented in MATLAB R2017a using the forward Euler method.

## 3. Results

### 3.1. Modulating the effect of SFA on output correlation by changing the firing rate

**Figure 3:**
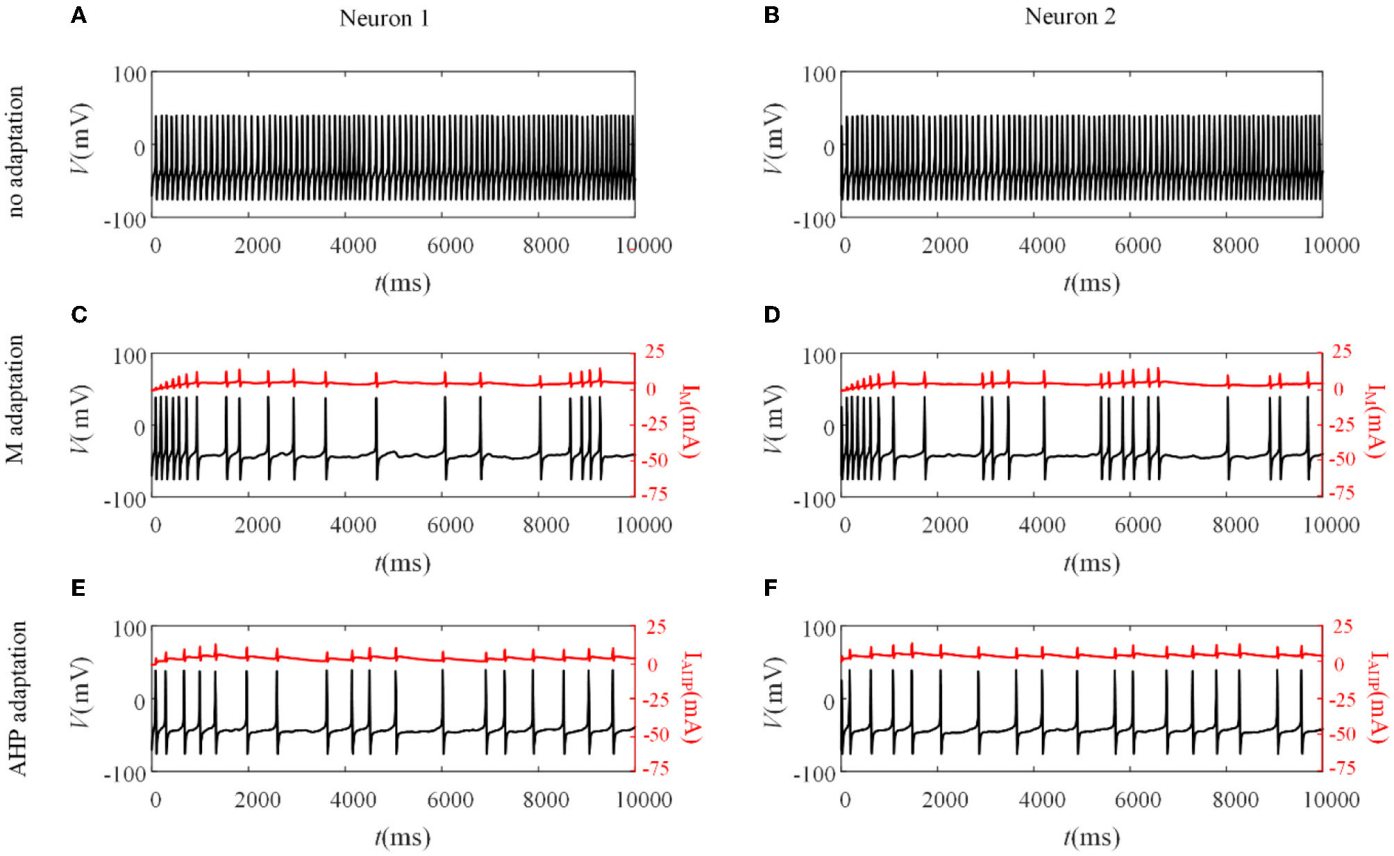
Variation of membrane potentials and adaptation currents with or without SFA. **(A, B)** The membrane potential of pairwise neurons under correlated input without adaptation. **(C, D)** The firing patterns and M current of pairwise neurons where $g_{M}=\text{0}\text{.25 mS}/\text{c}{\text{m}}^{\text{2}}$. **(E, F)** The firing patterns and AHP current of pairwise neurons where $g_{AHP}=\text{2}\text{.5 mS}/\text{c}{\text{m}}^{\text{2}}$.

Different adaptation mechanisms are applied to pairwise neurons to examine how SFA shapes the neuronal output response between pairwise neurons. The neuronal output response without the SFA is first explored, as depicted in Figures 3A, B. The firing patterns are sensitive to the correlated input, and the membrane potentials exhibited oscillations. The non-uniform distribution of the patterns resulted from noisy inputs. We then examined the neural activity with adaptation (M current or AHP current). As shown in Figures 3C, D, the M current makes the output responses sparser than those without adaptation. This slowing down of the firing activity also exists in the situation with the AHP current, as shown in Figures 3E, F. The addition of adaptation decreases the firing rate of individual neurons. The inhibitory effect of SFA on slowing the firing patterns might further decrease the output correlation.

**Figure 4:**
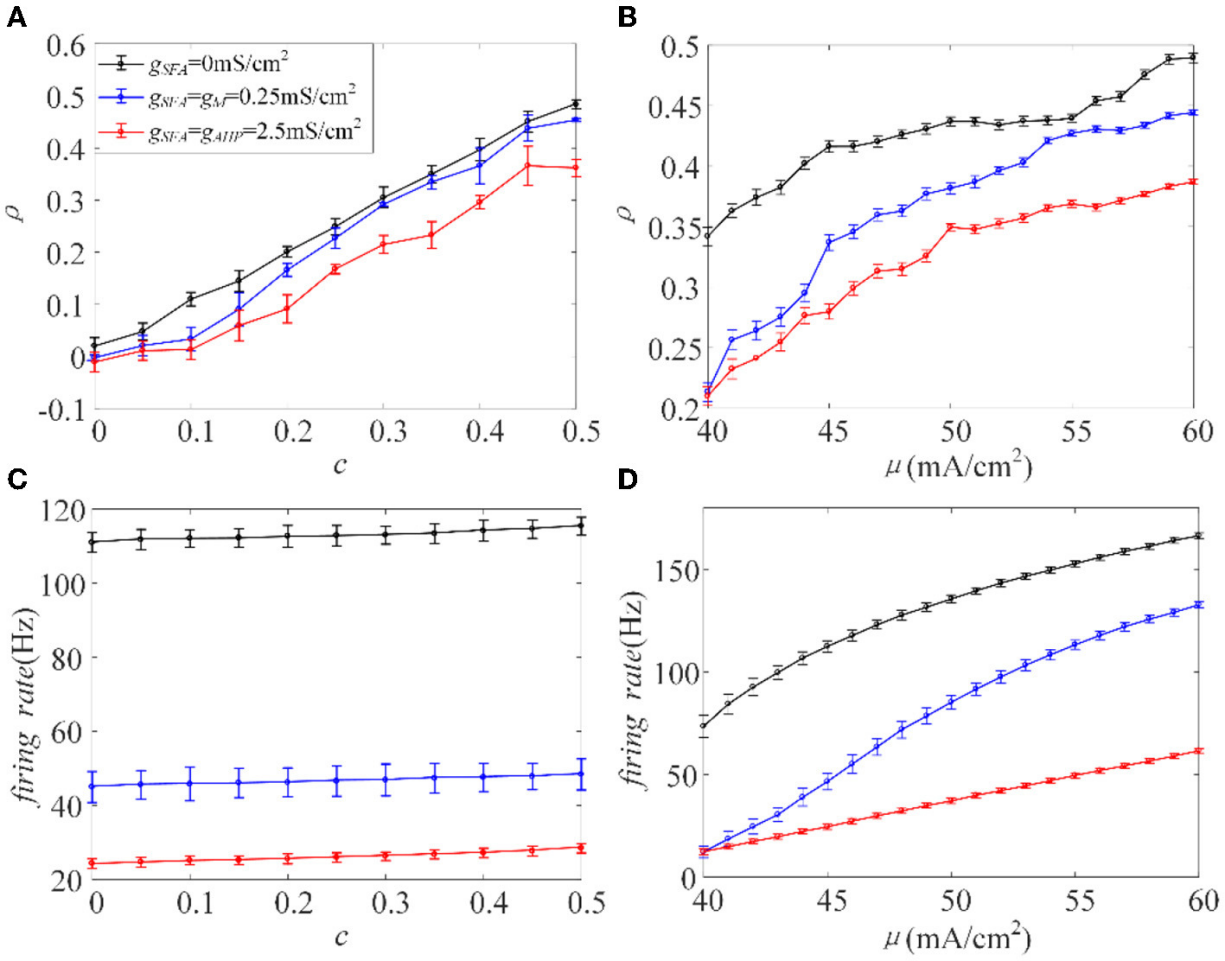
Relationship between output correlation and firing rate in the Prescott model $g_{M}=\text{0}\text{.25mS}/\text{c}{\text{m}}^{\text{2}}$, $g_{AHP}=2.\text{5mS}/\text{c}{\text{m}}^{\text{2}}$, σ^2^ = 5(mS/cm^2^)^2^. **(A)** The positive relationship between input correlation and output correlation. The output correlation presents a decrease in the case of adaptation μ = 45mA/cm^2^. **(B)** The output correlation varies with input intensity under different adaptation conditions *c* = 0.5. **(C)** The relationship between firing rate and input correlation μ = 45mA/cm^2^. **(D)** Firing rate vs. correlated input current (*f*−μ) variations. *c* = 0.5. τ_*T*_ = 400ms and τ_*s*_ = 50ms.

The inhibition generated by adaptation slows the spiking activity of single neurons. Next, we investigated how the inhibitory effect changes the output correlation ρ, using the input correlation *c*, and correlated input intensity μ. The variance of the correlated input is fixed at a constant value. The existence of adaptation made ρ less sensitive to the input correlation, where the slope of the variation slowed down, see Figure 4A. The value of ρ decreased with the addition of adaptation at the same *c* value. As the value of *c* ranged from 0 to 0.5 with an increase of 0.05, the output correlation and firing rate are calculated, as shown in Figures 4A, C, respectively. The output correlation for each adaptation is monotonic as *c* increases. The firing rate increases slowly with the enhancement of *c*, and the higher conductance intensity induces lower firing rate level. Because of the large ordinate space in Figure 4C, the change in firing rate with c is not obvious, but when viewed at higher resolution (not shown here), firing rate did show a significant increase with *c*. According to the above experiments, ρ values and the firing rate exhibited similar variation trends as the value of *c* increased. Therefore, we speculate that adaptation decreases ρ by decreasing the firing rate. We then studied the input intensity's influence on the output correlation ρ. As shown in Figures 4B, D, ρ and the firing rates are both sensitive to μ. The value of ρ increased while μ also increased, and the firing rate showed the same tendency under the same stimulus. The introduction of the M and AHP currents decreased the ρ value, and a decrease in the firing rate accompanied this. Combined with the above speculation of ρ and firing rates, we conclude that the M and AHP currents decrease the output correlation by decreasing the firing rate. The firing rate serves as a bridge connecting the SFA and output correlation, which causes the inhibition of the firing rate at the cellular level and decreases the output correlation at the network level.

### 3.2. Shaping output correlation via varying adaptation currents

**Figure 5:**
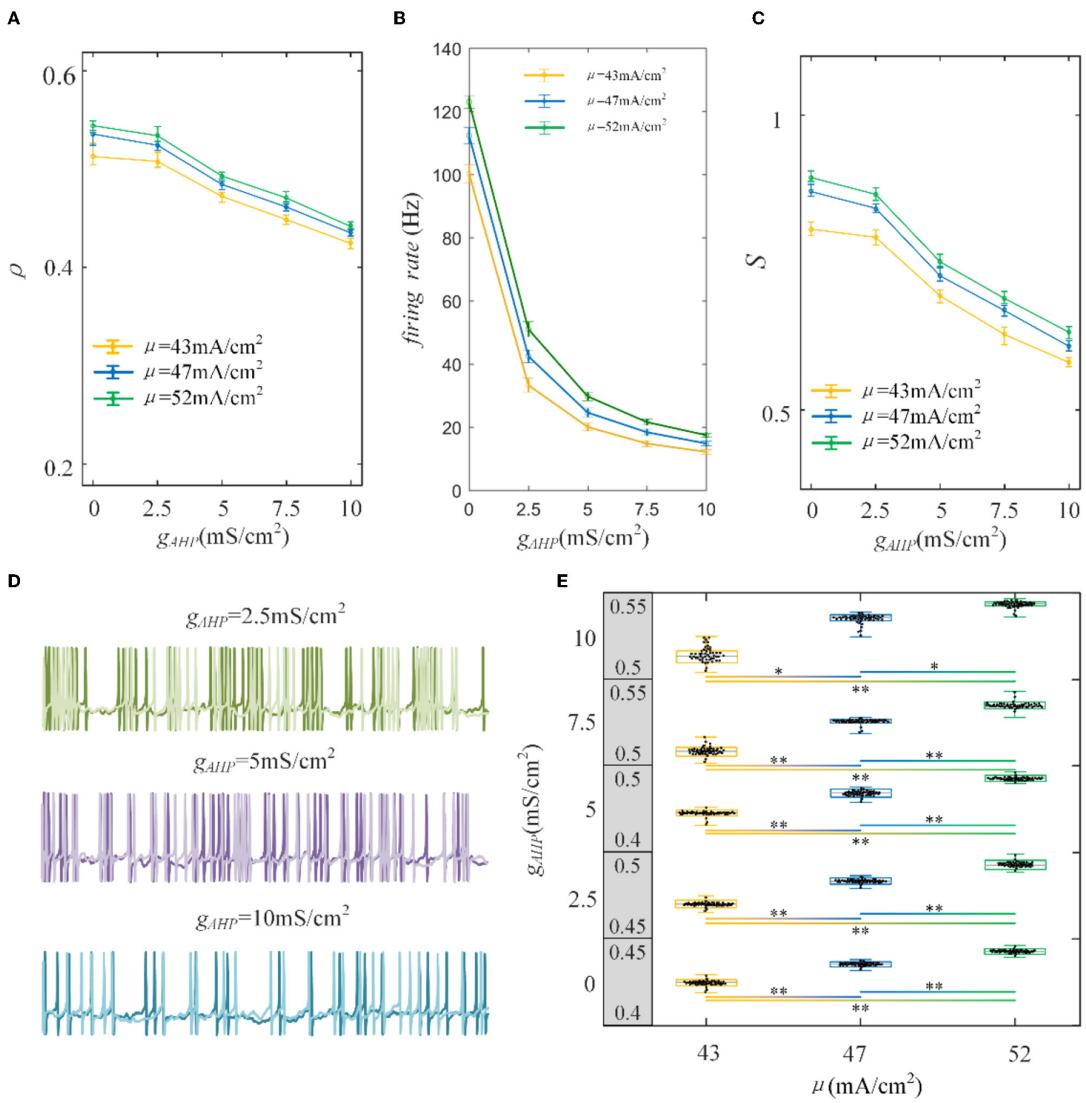
Correlation analysis vs. AHP adaptation conductance and three types of input intensity. **(A)** The correlation decreases with the increase in AHP adaptation conductance. The correlation shows a downward trend. For the same *g*_*AHP*_, the correlation enlarges as the input current increases. **(B)** The tendency of the firing rate with the enhanced AHP conductance. The decreasing tendency is gentle under different correlated inputs. **(C)** The susceptibility vs *g*_*AHP*_. The susceptibility decreases with increasing *g*_*AHP*_. As the input intensity enhances, the susceptibility enlarges. **(D)** The spike trains under μ = 43mA/cm^2^. The length of the sequence is 1,000 ms. **(E)** The two-sample *t*-test is utilized to test the difference between different input intensities. The gray area presents the output correlation. The numbers represent the upper and lower bounds of output correlation. The results of 50 trials are shown in the boxplot. The results show that different intensities under the same *g*_*AHP*_ generate significantly different output correlations. *Presents *p* < 0.01. **Presents *p* < 0.001. *c* = 0.6. τ_*T*_ = 400ms and τ_*s*_ = 50ms.

To examine how the adaptation current shapes the neuronal output response, we varied the adaptation conductance of the M and AHP currents. The intensity of the adaptation current depends on conductance, which is directly related to the firing rate of the neurons. A stronger adaptation current generates a lower firing rate. As shown in Figure 5A, the correlation decreases significantly with the increase in AHP adaptation conductance compared with the correlation without the SFA. The output correlation slows as the AHP conductance increases. In addition, the enhancement of input enlarges the output correlation generally. Three examples of firing sequences are superimposed with different colors, as listed in Figure 5D. It is obvious that the spike trains of the pairwise neurons become sparse. At $g_{AHP}=2.5\;\text{mS/c}{\text{m}}^{\text{2}}$, the firing is swift at the beginning. Then the spike train follows with slow sparse spikes. As *g*_*AHP*_ increases to 5 mS/cm^2^ and 10 mS/cm^2^, the rapid firing becomes less noticeable at the initial stage. Furthermore, the spike train correlation becomes weakened. The sparse spiking reflects the slowing firing rate, as shown in Figure 5B. The firing rate tended to decrease as *g*_*AHP*_ increases. The correlation variation corresponds to the firing rate variation, and they present the same decreased variation tendency as *g*_*AHP*_ enhances. Under AHP current, the firing rate variation presents a gentle decreased tendency, which is corresponded to the correlation. This slowing effect changes the v of the output correlation to the input correlation. The susceptibility is displayed in Figure 5C. The results show that the susceptibility decreases as *g*_*AHP*_ increases. The variation trend of susceptibility is consistent with the output correlation. The enhancement of spike frequency adaptation decreases the interaction of pairwise neurons, which further attenuates the susceptibility. Furthermore, the significant difference between different input intensities is investigated to demonstrate the role of input intensity on output correlation, as shown in Figure 5E. The results show that there is a significant difference between different inputs. The external inputs play a pivotal role on output correlation. The enhancement of external input intensity enlarges the correlation apparently.

**Figure 6:**
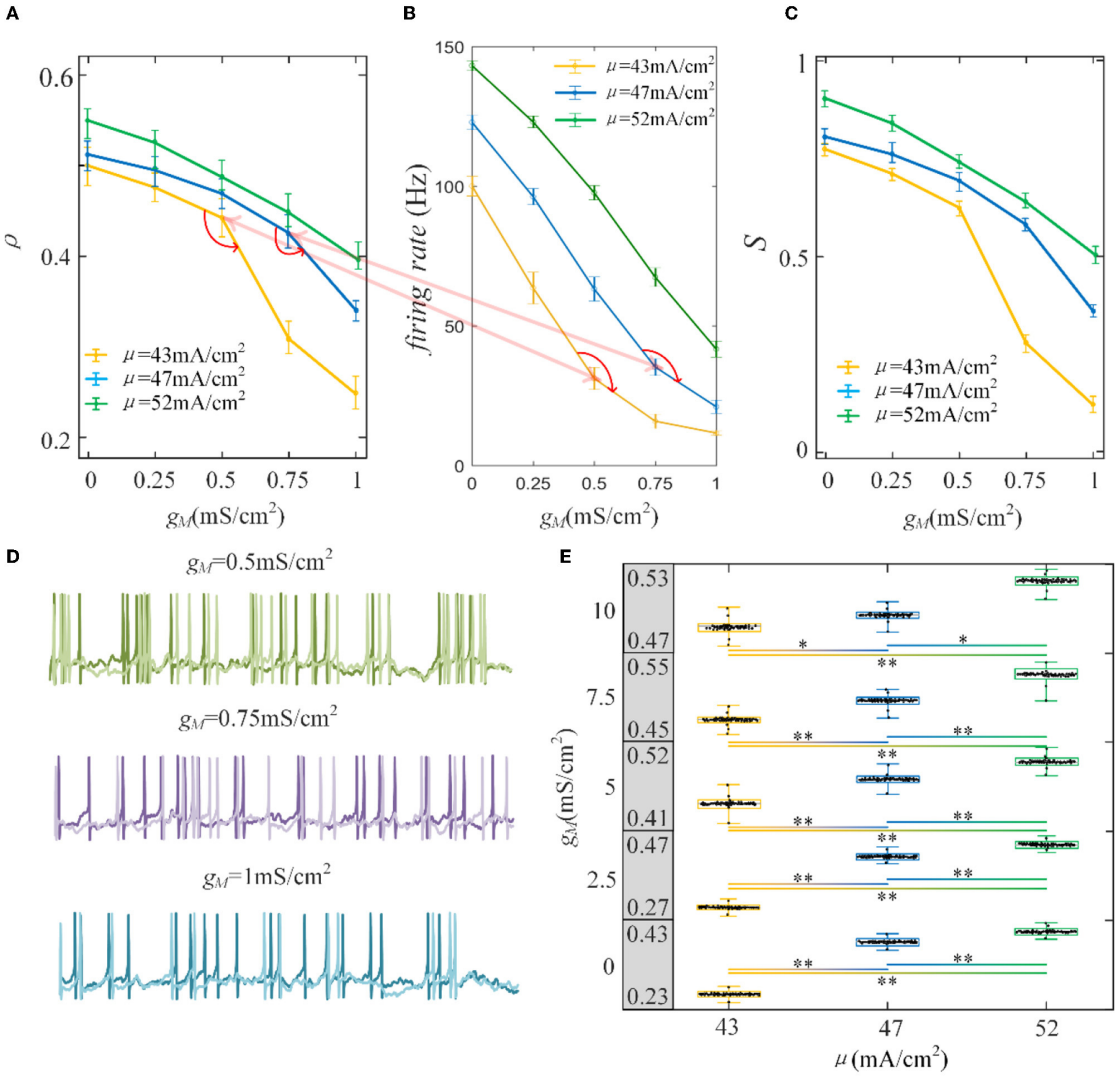
Correlation analysis vs. M adaptation conductance and three types of input intensity. **(A)** The output correlation vs. M adaptation conductance. ρ decreases smoothly as/increases. **(B)** The firing rate variation with M conductance. Large angles appear at $g_{M}=0.75\text{mS/c}{\text{m}}^{\text{2}}$ with μ = 47mA/cm^2^ and $g_{M}=0.5\text{mS/c}{\text{m}}^{\text{2}}$with μ = 52mA/cm^2^. The firing rate modulation is the same as that in **(A)**. **(C)** The susceptibility vs. *g*_*M*_. The susceptibility decreases with increasing *g*_*M*_. As the input intensity enhances, the susceptibility enlarges. **(D)** The spike trains under μ = 43mA/cm^2^. The length of the sequence is 1,000 ms. **(E)** The two-sample *t*-test is utilized to test the difference between different input intensities. The gray area presents the output correlation. The numbers represent the upper and lower bounds of output correlation. The results show that different intensities under the same *g*_*M*_ are significant intensities. *Presents *p* < 0.01. **Presents *p* < 0.001. *c* = 0.6. τ_*T*_ = 400ms and τ_*s*_ = 50ms.

Furthermore, the role of M current on output correlation is investigated. As shown in Figure 6A, the output correlation attenuates as M conductance increases. During μ = 52 mA/cm^2^, the neuronal firing rate is sensitive to the mean value of the input. However, in terms of μ = 47 mA/cm^2^ and μ = 43mA/cm^2^, the correlation is significantly reduced, beginning with the red curved arrows. The corresponding spike trains are shown in Figure 6D. The firing patterns demonstrate an evident sparse distribution as *g*_*M*_ increases. From $g_{M}\text{=}0.5\text{mS}/\text{c}{\text{m}}^{2}$ to $g_{M}\text{=}0.75\text{mS}/\text{c}{\text{m}}^{2}$, the firing patterns present are sparser, which indicate that the firing rate is significantly reduced. As *g*_*M*_ enhances displayed in Figure 6B, the firing rate presents an obvious subdued tendency. The firing rate is relatively low and sensitive to input fluctuations, as shown in Figure 6C. In contrast to the AHP current, although the M current reduced the output correlation, the correlation variation is not smooth. This is because the interplay between the M current and the input intensity slows the firing rate to a minimal value. While for M current, the firing rate generates obvious variations at $g_{M}=0.75\;\text{mS/c}{\text{m}}^{\text{2}}$ with μ = 47 mA/cm^2^ and $g_{M}=0.5\;\text{mS/c}{\text{m}}^{\text{2}}$ with μ = 52 mA/cm^2^. These significant variations are corresponding to the correlation variation in Figure 6B. The firing rate variation results in the correlation variation. In addition, the firing rate further decreases the susceptibility of input to output correlation, as displayed in Figure 6C. As shown in Figure 6E, the two-sample *t*-test indicates that the correlation at the same adaptation conductance under different inputs is significantly difference. The variation of susceptibility is consistent with the trend of output correlation. In conclusion of the role of SFA in output correlation, the correlated input and adaptation conductance determined the firing rate, and the output correlation is further modulated by the firing rate generated by the combined effect. The decreased firing rate depends on the attenuated SFA conductance and further inhibits the output correlation.

In the absence of the SFA, the maximum correlation value generated by the correlated input is approximated by the input correlation, which is consistent with previous findings (de la Rocha et al., 2007; Litwin-Kumar et al., 2011). Prescott et al. (2006) found a terminated firing phenomenon (i.e., the cessation of spiking activity) that causes firing patterns to be generated only at the onset of a stimulus (Prescott and Sejnowski, 2008). In our simulations, this terminated firing phenomenon 'appears' but the noise component of correlated input still generates some sparse firing patterns as depicted in Figure 6D. It is worth mentioning that the form of the terminated firing phenomenon is controlled by two factors: stimulus intensity and adaptation conductance. Low stimulus intensity and adaptation conductance generate the terminated firing phenomenon more easily. This phenomenon causes the sensitivity of the firing rate to change from the input intensity to the noise component of the input, resulting in a significant decrease in the firing patterns, which become very sparse. In conclusion, different SFA mechanisms have various effects on output correlation. However, they control the correlated input or adaptation conductance to change the firing rate and have a decreased impact on output correlation.

**Figure 7:**
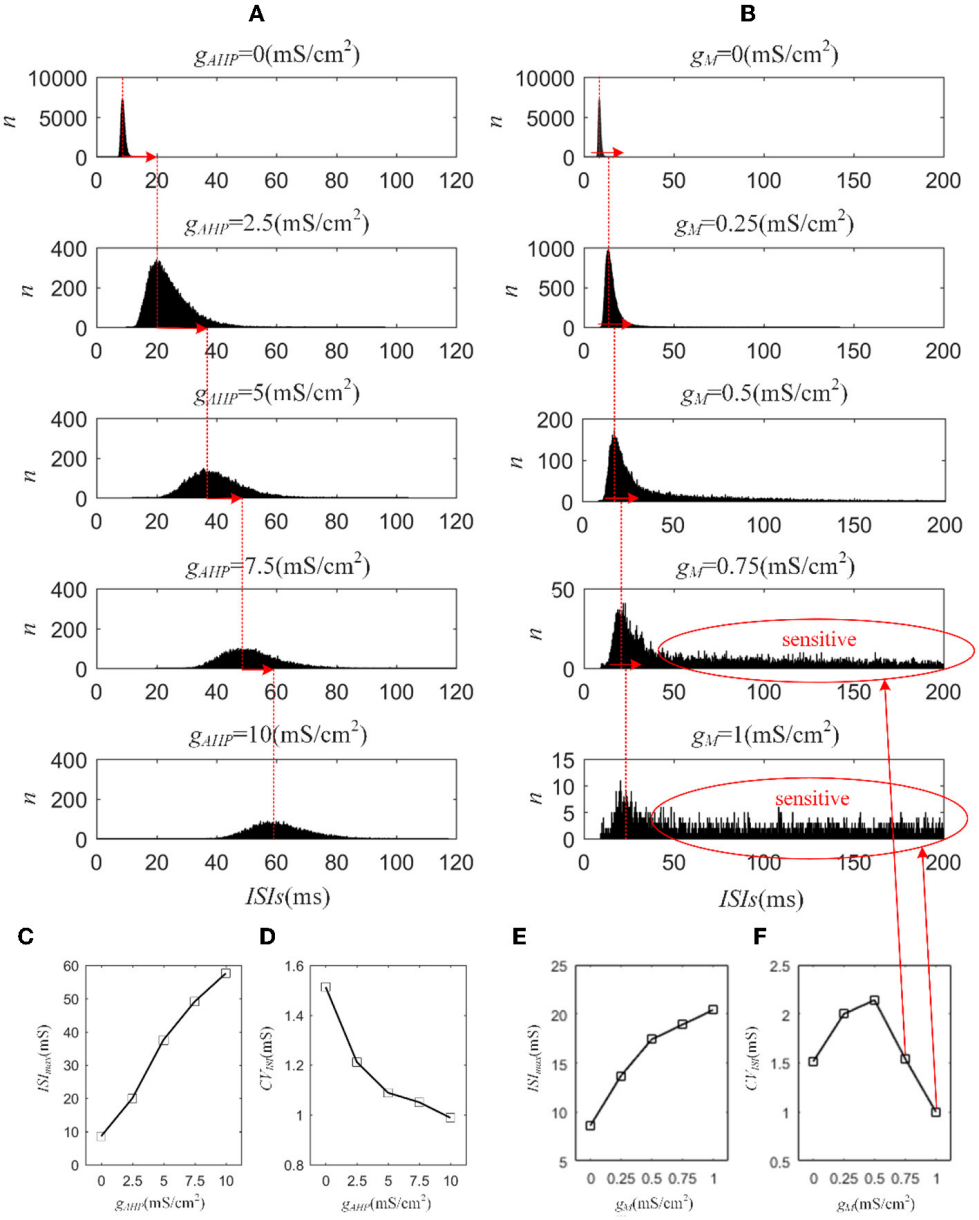
Distribution of *ISIs* with M current or AHP current μ = 43mA/cm^2^. **(A)** *ISI* distributions in the case of AHP current. The mountainous area gradually moves to the right part as *g*_*AHP*_ increases. **(B)** *ISI* distributions in the case of M current. **(C)** The distribution of *ISIs* with the largest number under different AHP adaptation conductance. **(D)** The *CV* of the *ISIs* shows a smooth decrease. **(E)** The distribution of *ISIs* with the largest number under different M adaptation conductance. **(F)** An inflection point appears at $g_{M}\text{=}\text{0}\text{.5mS}/\text{c}{\text{m}}^{\text{2}}$ because the firing patterns show an obvious decrease τ_*T*_ = 400ms and τ_*s*_ = 50ms.

Next, we investigated how adaptation affected this correlation. To quantify the different effects of the M current and AHP current, the *ISIs* and their histograms are calculated. The *ISI* histograms, under different adaptation conductance, are shown in Figure 7. The *ISIs* without SFA are distributed at lower values ~10 ms, with a substantial peak. In this case, pairwise neurons displayed a rapid firing process. As the adaptation conductance increases, the adaptation currents gradually increase, decreasing the firing rate. The diagrams with the AHP current in Figure 7A show a regular change: ISIs are distributed over a broader range on the horizontal scale. The values of the *ISIs* along the x-axis are increasing with increasing adaptation conductance, while the amount of *ISIs* reduces. The variations in the *ISIs* in Figure 7B with the M current are similar to those with the AHP current. It is worth noting that there is a significant difference: ISIs with AHP current show a stable change from top to bottom as shown in the subgraph of Figure 7B, whereas *ISIs* with M current show an evident variation from $g_{M}\;\text{=}\;0.5\text{mS}/\text{c}{\text{m}}^{2}$ to $g_{M}\;\text{=}\;0.75\text{mS}/\text{c}{\text{m}}^{2}$ because this adaptation is sufficient to make the firing patterns sparse. The number of *ISIs* decreases dramatically, and larger values are gradually generated by the noisy component of the input, which becomes a dominant factor in changing the correlation. The large values of *ISIs* become essential in decreasing the firing rate and further reducing the output correlation when the adaptation conductance increases. As shown in Figures 7C, E, the mean value of the *ISIs* increased with an increase in the adaptation conductance. The increase in Figure 7C is relatively uniform, whereas the variation trend in Figure 7E slows at $g_{M}\;\text{=}\;0.5\text{mS}/\text{c}{\text{m}}^{2}$. This is because the AHP current decreases the firing rate slightly, whereas the M current generates a significant decrease in the firing rate at $g_{M}\;\text{=}\;0.5\text{mS}/\text{c}{\text{m}}^{2}$. Meanwhile, the coefficient of variation of the ISIs at $g_{M}\;\text{=}\;0.5\text{mS}/\text{c}{\text{m}}^{2}$ increases at the inflection point. For $g_{M}>0.5\text{mS}/\text{c}{\text{m}}^{2}$, the firing patterns are generated by correlated noise, which causes the firing patterns to be generated randomly, and the firing becomes more irregular. Under adaptation conductance where $g_{M}>0.5\text{mS}/\text{c}{\text{m}}^{2}$, the *CV* of the *ISIs* no longer increases but shows a decreasing trend. This trend is consistent with the variation shown in Figure 6A. In conclusion, AHP and M adaptation decrease the output correlation with an increase in the strength of the adaptation currents. The attenuation in output correlation is accompanied by the increment in ISIs distribution.

### 3.3. Effect of adaptation conductance and stimulation time on output correlation

**Figure 8:**
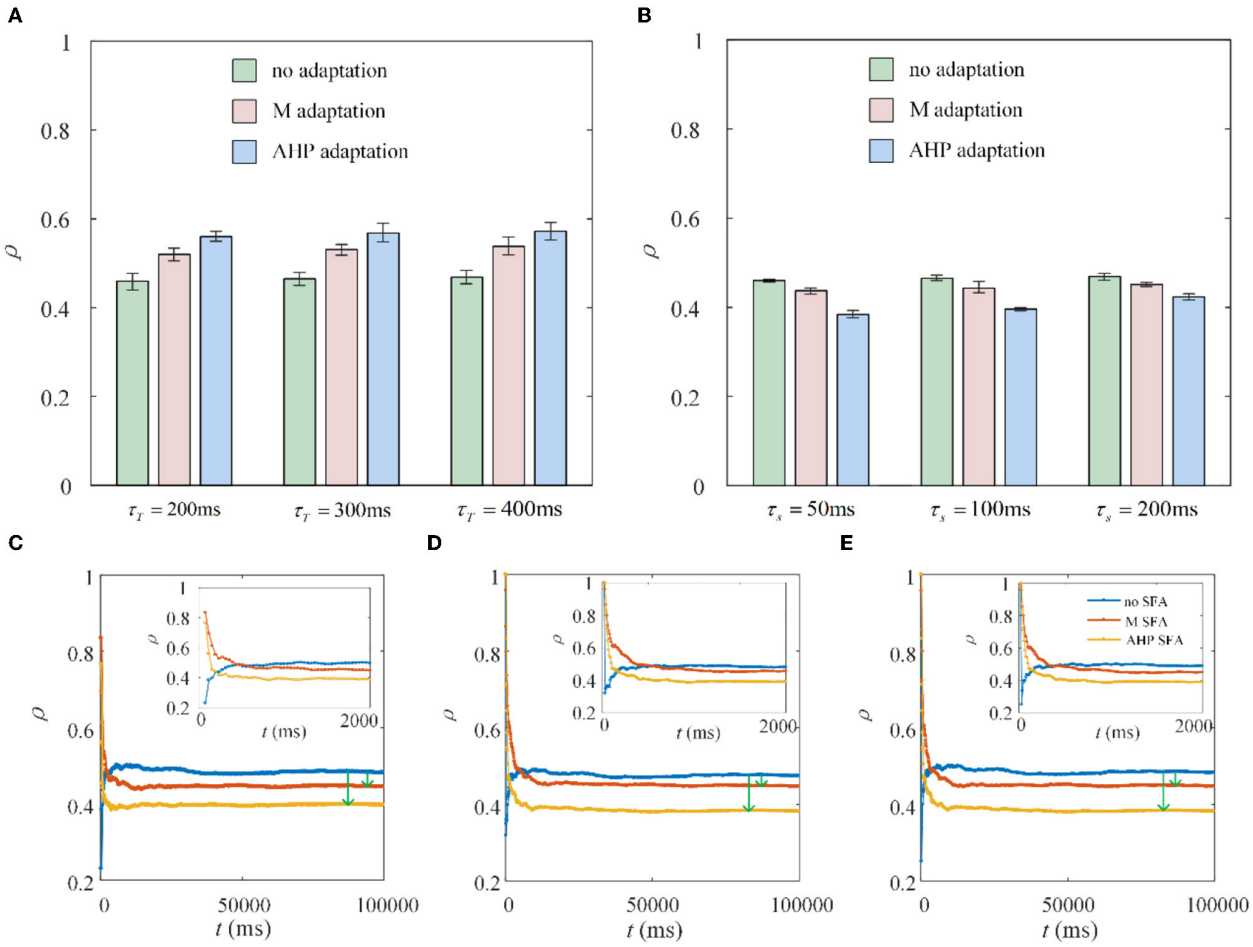
Correlation coefficient ρ vs. time window τ_*T*_ and slide window τ_*s*_. **(A)** The correlation variation with stimulation duration *t* fixed at 1, 000ms. τ_*s*_ = 100ms. Compared with the correlation without SFA, the output correlation presents an increase with M adaptation or AHP adaptation. **(B)** The correlation variation with stimulation time fixed at 10, 000ms. The correlation suggests a decrease with SFA τ_*T*_ = 400ms. **(C)** The correlation varies with stimulation time where τ_*T*_ = 400ms and τ_*s*_ = 50ms. **(D)** The correlation varies with stimulation time where τ_*T*_ = 300ms and τ_*s*_ = 100ms. **(E)** The correlation varies with stimulation time where τ_*T*_ = 400ms and τ_*s*_ = 100ms.

The dynamic firing process of neurons determines the variation in the special output correlation, while the output correlation depends on the firing rate for a given fixed shared input. There are two stages of firing rate variations in the situation with SFA. From a single neuron point of view, in the first stage, the neurons show rapid firing as shown in the front part of Figure 1G. While in the second stage, the neurons exhibit slow and steady firing. We discovered an interesting phenomenon under the same parameter settings, except for the stimulation time. The experimental results are the opposite. The stimulation times in Figures 8A, B are set to 1,000 and 10,000 ms, respectively. As shown in Figure 8A, the correlation increases because the rapid firing rate in the first stage dominates the correlation. As the stimulation time increases to 10,000 ms, the effect of rapid firing weakens, and the slow firing process in the second stage primarily impacts the correlation. The question then arises as to how long it would take for the stimulation time to generate a stable reduction in the correlation. To explore this question, we randomly selected three combinations of time (τ_*T*_) and slide windows (τ_*s*_). As described in Figures 8C–E, the correlation presents a trend of increasing followed by decreasing. The variation in correlation arises from the changing rules of the firing rate with the SFA. The output correlation shows a temporary increase for a very short stimulation time, as shown in the upper-right corner of each subgraph in Figures 8C–E. This is due to the rapid firing rate, resulting in shared spike trains of pairwise neurons having more similar parts in the initial stage. However, this immediate increase in the correlation disappeared and is reversed to a decreased result when the stimulation time increased. In the second stage, the SFA decreased the correlation by slowing down the firing rate of individual neurons. The slow firing patterns in the second stage may have an important impact on the population coding of sustained input signals to the neurons.

**Figure 9:**
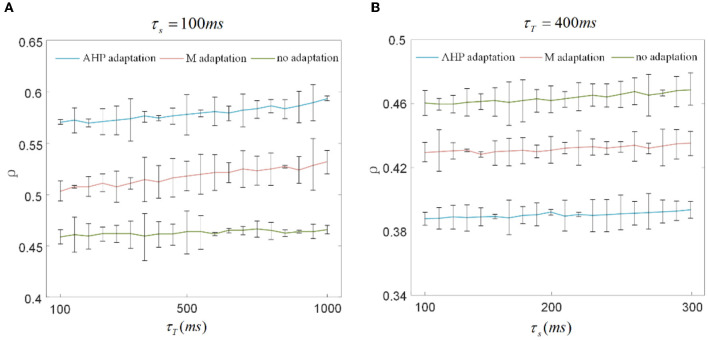
Correlation variation with stimulation time fixed to 1, 000ms and 10, 000ms. **(A)** The correlation variation with τ_*s*_ fixed at 100ms. τ_*T*_ varies from 100ms to 1, 000ms. The correlation with SFA demonstrates an increase compared to that without adaptation. **(B)** The correlation variation with τ_*s*_ varying from 100ms to 300ms where τ_*T*_ is set to 400ms. The correlation presents a decrease under the corresponding parameter settings.

The output correlation is measured by Pearson's coefficient, resulting in the output correlation being related to τ_*T*_ and τ_*s*_ (Barreiro et al., 2012). Because only a fraction of spike trains is included in τ_*T*_, the instantaneous partial firing rate may generate significant variability in the output correlation (de la Rocha et al., 2007; Barreiro et al., 2012). Different combinations of τ_*s*_ and τ_*T*_ are selected to verify whether they affected the decreased results of SFA on the correlation in this study. The chosen time window in this section is 10 times larger than the slide window (Barreiro et al., 2012). As shown in Figure 9A, τ_*s*_ is fixed at 100 ms to observe the effect of τ_*T*_ on output correlation. The output correlation exhibits a weak increase across the range of values of τ_*T*_. Similar to the variation tendency in Figure 5A, the correlation variations caused by τ_*s*_ are not noticeable when the stimulation time is fixed at 10,000 ms in Figure 9B. All results show that τ_*s*_ and τ_*T*_ have an impact on the numerical value of the correlation. However, their different combinations make no difference to the effect of SFA on reducing the correlation. Stimulation time is the most critical factor affecting the correlation difference between the two graphs in Figure 8. High similarity in the initial part of the spike trains with the SFA significantly affects the correlation for a short stimulation time. The correlated input and the input correlation become the main factor decreasing the correlation over a long stimulation time.

### 3.4. Verifying the effect of different adaptation mechanisms on decreasing correlation

M and AHP currents have been proven to reduce the output correlation by decreasing the firing rate in this study. The Prescott model is a single-compartment conductance-based physical model that neglects other adaptations of the biological model, such as the *Na*^+^ inactivation *K*^+^ current. In this section, we explore the effect of the slow inhibitory current (*I*_*KNa*_) on this correlation. In addition to the adaptation current, the dynamic threshold can generate adaptation and decrease the firing rate.

**Figure 10:**
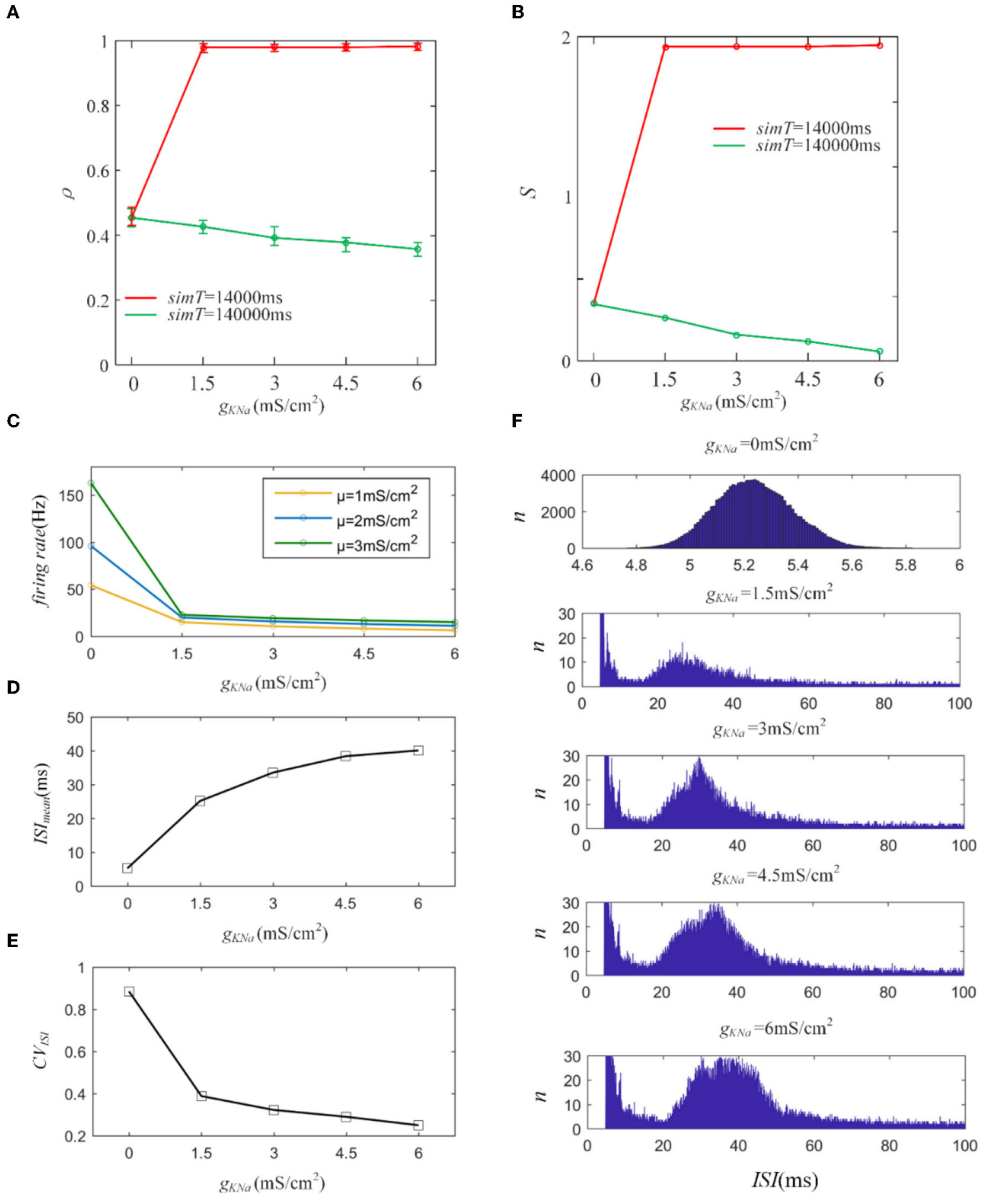
**(A)** Variation of output correlation with different *g*_*KNa*_ in two different stimulation times. The stimulation time of the red and green lines is 14, 000ms and 140, 000ms, respectively. The red line presents an increase due to the increased firing rate, while the green line demonstrates a decrease because of the decreased firing rate. **(B)** The susceptibility variation with the increased *g*_*KNa*_. **(C)** The firing rate variation under different *g*_*KNa*_. **(D)** The mean value of *ISI* with different conductance levels. **(E)** The coefficient of variation of the *ISIs*. **(F)** The variation of *ISIs* with different adaptation conductance levels. With the enlargement of the adaptation conductance, *ISIs* shift to the right region of the horizontal axis. *c* = 0.6. τ_*T*_ = 400ms and τ_*s*_ = 50ms.

First, we introduce a typical *I*_*KNa*_ from the Wang model to explore its effect on output correlation (Wang et al., 2003). The red line in Figure 10A indicates an increase in the output correlation, completely different from the decreasing phenomenon in Section 3.1. The sequences generate rapid firing for a long time, even more than 3,000 ms, which makes the front of the spike trains highly similar. The similarity is hardly affected by the random spiking activity in the correlated input. Therefore, the correlation remains very high, close to 1. The firing rate decreased, resulting in the correlation demonstrating a steady decline as the adaptation conductance increased, as shown in Figure 10B. This reduced effect of the adaptation current on the correlation is consistent with the above results for M and AHP currents. We further studied the variation in *ISIs*. The *ISIs* exhibited a regular hill without an SFA. As the adaptation conductance increased, the *ISIs* hill moved toward larger values, as depicted in Figure 10E. This hillside is a significant factor that decreased the correlation. We then calculated the mean value of the *ISIs* that exhibited a gentle ascent, as shown in Figure 10C. Because the fluctuations in the correlated input dominated the correlation variation, the *CV* of the *ISIs* demonstrated a decreasing tendency, as shown in Figure 10D. In conclusion, *I*_*KNa*_ decreased the correlation by decreasing the firing rate.

**Figure 11:**
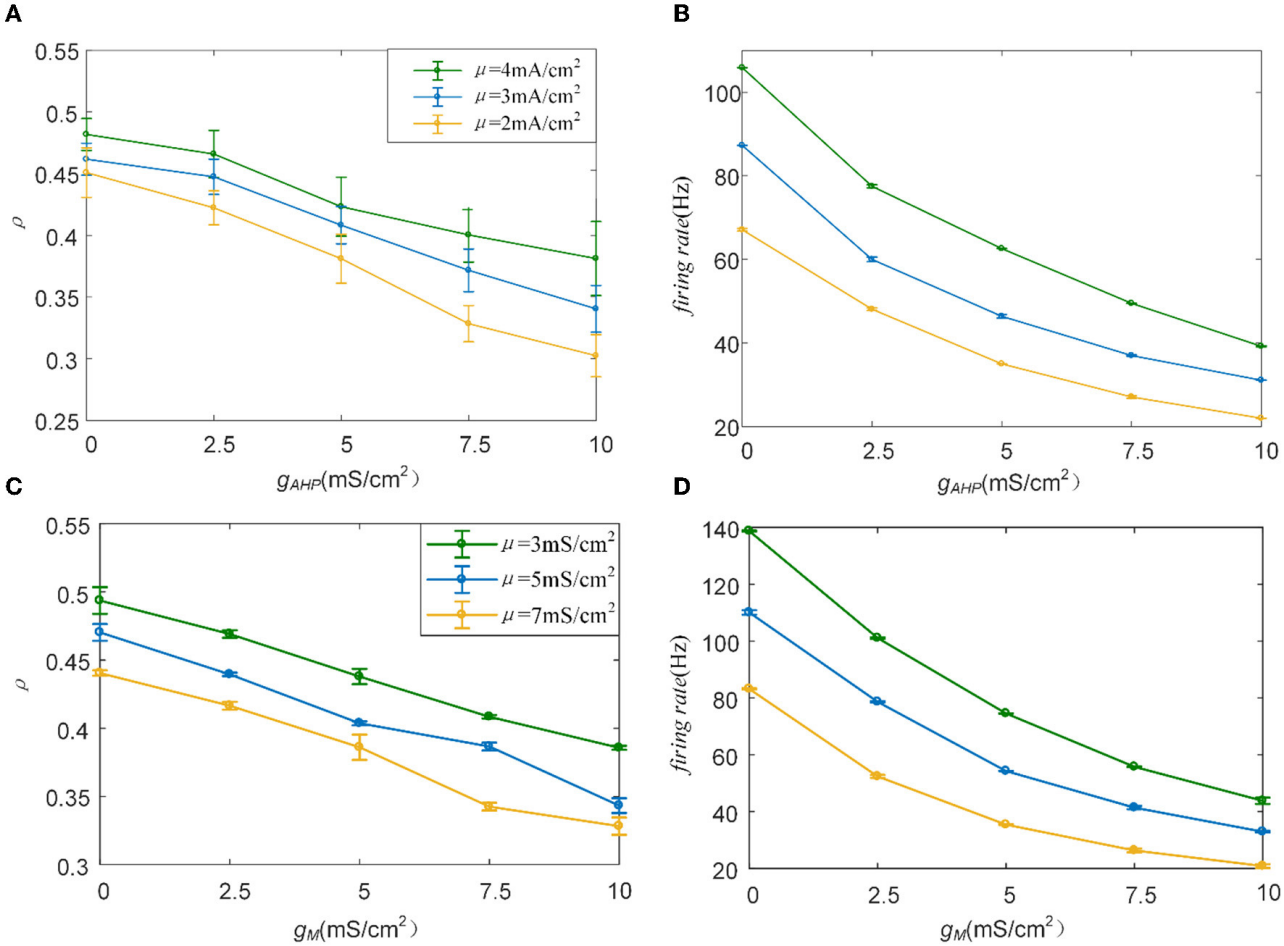
Variation of correlation and firing rate as conductance changes. **(A)** The correlation vs. conductance of AHP. **(B)** The firing rate vs. conductance of AHP. **(C)** The correlation vs. conductance of M. **(D)** The firing rate vs. conductance of M. *c* = 0.6. τ_*T*_ = 400ms and τ_*s*_ = 50ms.

**Figure 12:**
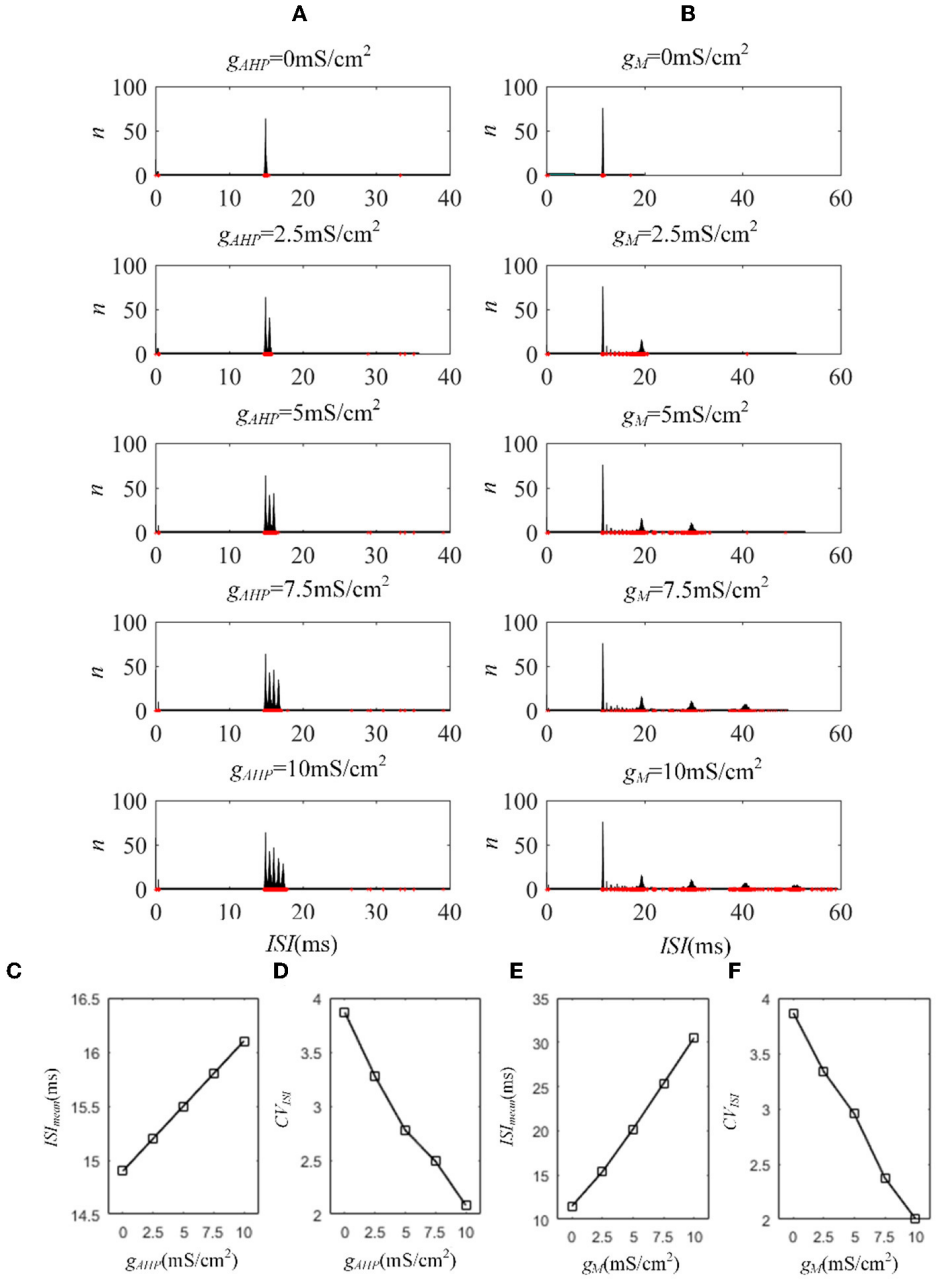
*ISI* variation of the Ermentrout model with AHP and M currents. **(A)** *ISI* variations under different adaptation conductances, under AHP current. The blue points represent the *ISIs*. **(B)** *ISI* variations with different adaptation conductances under M current. **(C)** The mean value of *ISIs* with different conductances under AHP current. **(D)** Coefficient of variation of the ISIs under AHP current. **(E)** The mean value of ISI with different conductances under M current. **(F)** Coefficient of variation of the ISIs under M current. *c* = 0.6. τ_*T*_ = 400ms and τ_*s*_ = 50ms.

The M and AHP currents have been proven to decrease the correlation in the Prescott model. However, the same mechanisms may behave differently in different models. We chose the Ermentrout model to verify whether the same SFA mechanisms in the various models had diverse effects on the correlation. Two aspects confirm that the results are independent of the models: the density of the input current and the adaptation conductance. As shown in Figure 11A, the correlation decreases as the adaptation conductance increases. At the same conductance, increasing the input intensity enhanced the output correlation. SFA mechanisms are the primary factors that change the correlation by decreasing the firing rate, as displayed in Figure 11B. Different SFA mechanisms in various models generated similar reduced effects on the correlation. The most remarkable difference between the Ermentrout and the Prescott models is that the correlation (Figure 11C) and firing rate (Figure 11D) in the Ermentrout model reduces smoothly as compared to the correlation change seen with the Prescott model. The downward trend of the output correlation curves is smoother without inflections. The correlation demonstrates continuous variation as *g*_*AHP*_ increases. The variation in firing patterns with AHP current is a continuous change with adaptation conductance changes. Thus, the variation in correlation presents a smooth decrease regardless of whether in the Prescott or Ermentrout model. The decreased correlation is caused by a reduction in firing rate, accompanied by *ISIs* variations. The *ISIs* are plotted in Figure 12, which shows that the distribution expands to larger values. In contrast to the variation in the Prescott model, the Ermentrout model is more regular because the firing activity does not stop during the response.

**Figure 13:**
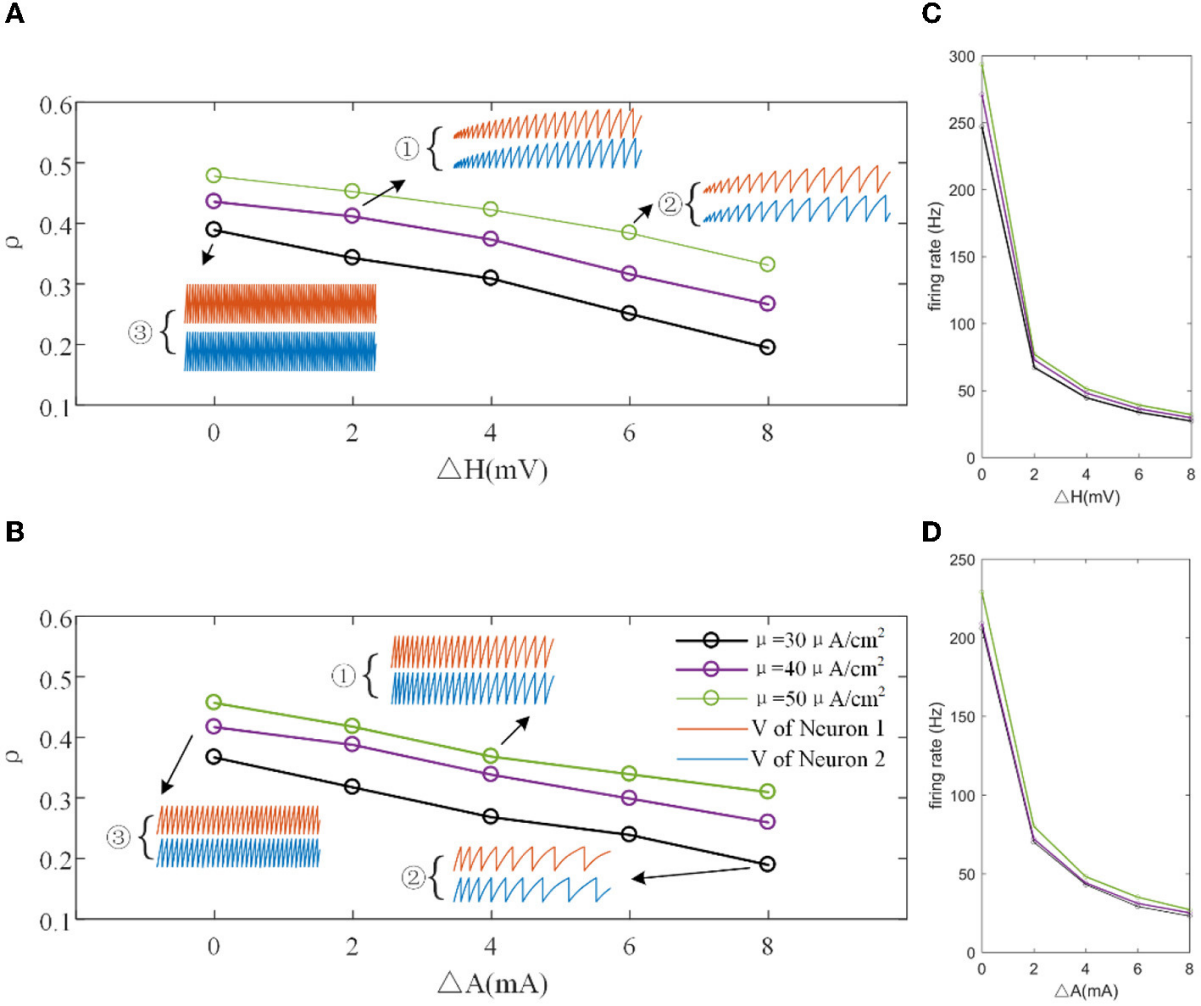
**(A)** Correlation variation with different input current intensities in the LIFDT model. ①, ②, and ③ are the firing patterns for the first 1,000 ms of stimulation. **(B)** The correlation variation with different input threshold intensities in the LIFAC model. **(C)** The firing rate vs. ΔH. **(D)** The firing rate vs. ΔA. *c* = 0.6. τ_*T*_ = 400ms and τ_*s*_ = 50ms.

**Figure 14:**
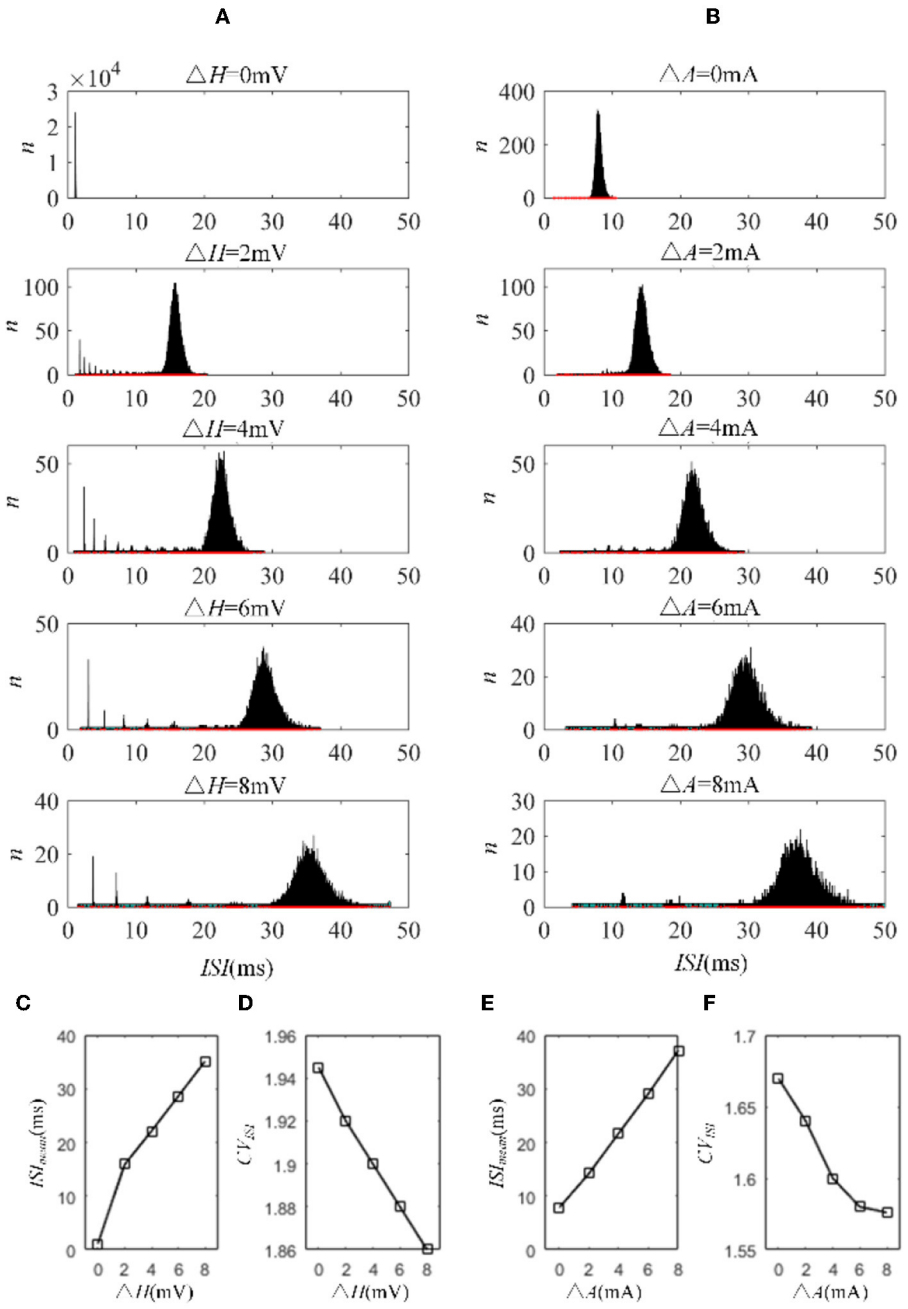
*ISI* variation in LIFDT and LIFAC models. With the increasing adaptation current in the left panel or dynamic threshold in the right panel, the distribution of *ISIs* shows a shift to a higher value on the abscissa and a decline in the amount. **(A)** *ISI* variations in the LIFDT model. **(B)** ISI variations in the LIFAC model. **(C)** The mean value of *ISIs* with different conductances under AHP current. **(D)** *CV* of *ISIs* under AHP current. **(E)** The mean value of *ISI*s with different conductances under M current. **(F)** *CV* of *ISIs* under M current. *c* = 0.6. τ_*T*_ = 400ms and τ_*s*_ = 50ms.

The mechanisms related to SFA introduced above are based on the current mechanism in a physiological phenomenon. Different ionic currents generate various adaptation phenomena. The models all verified that the effect of SFA is to reduce output correlation. This brings to question whether this SFA effect exists in a mathematical, physical model where dynamic thresholds generate SFA. First, we use LIFAC to generate adaptation current and verify whether adaptation current has the effect of decreasing correlation. Then, we test this decreased effect with another model using a dynamic threshold. As shown in Figure 13B ③, the pairwise neurons present a relatively uniform and regular firing pattern without adaptation. The addition of adaptation current makes the firing patterns slow down (Figure 13B ① and ② ). As shown in Figure 13C, the firing rate becomes slower as the threshold increases. We speculate that the adaptation current decreases the correlation by reducing the firing rate. When the dynamic thresholds are applied to the model, the membrane potential fluctuations become sparse, where the membrane potential shows dense distribution at the beginning and then slows down. With the increase in dynamic thresholds, the correlation presents a downward trend; see Figure 13A. Meanwhile, the firing rate (Figure 13D) shows a similar decrease tendency to the correlation in Figure 13C. This variation reflects that the dynamic threshold changes the correlation by decreasing the firing rate. At the same stimulus intensity, a larger adaptation strength generates a smaller correlation resulting from the decreased firing rate and sparse firing patterns. Furthermore, increased input current intensity enlarges the output correlation under each dynamic threshold but is uninfluential to the decreasing correlation tendency. The pairwise neurons with adaptation current or dynamic threshold decrease the correlation when SFA is introduced. The firing rate presents a decrease in the same trend as shown in Figures 14C, D. In addition, the *ISI* distributions in Figures 14A, B prove that the increase of *ISIs* accompanies the decrease in correlation. The mean values of *ISIs* show a smooth ascent in Figures 14C, E, indicating an obvious decrease in the firing rates. On the contrary, the *CV* of *ISIs* shows a reduction in Figures 14D, F due to the correlated noise components gradually playing a more critical role in generating firing patterns. These results are in accordance with the previous results in Section 3.2.

### 3.5. A mechanistic analysis

**Figure 15:**
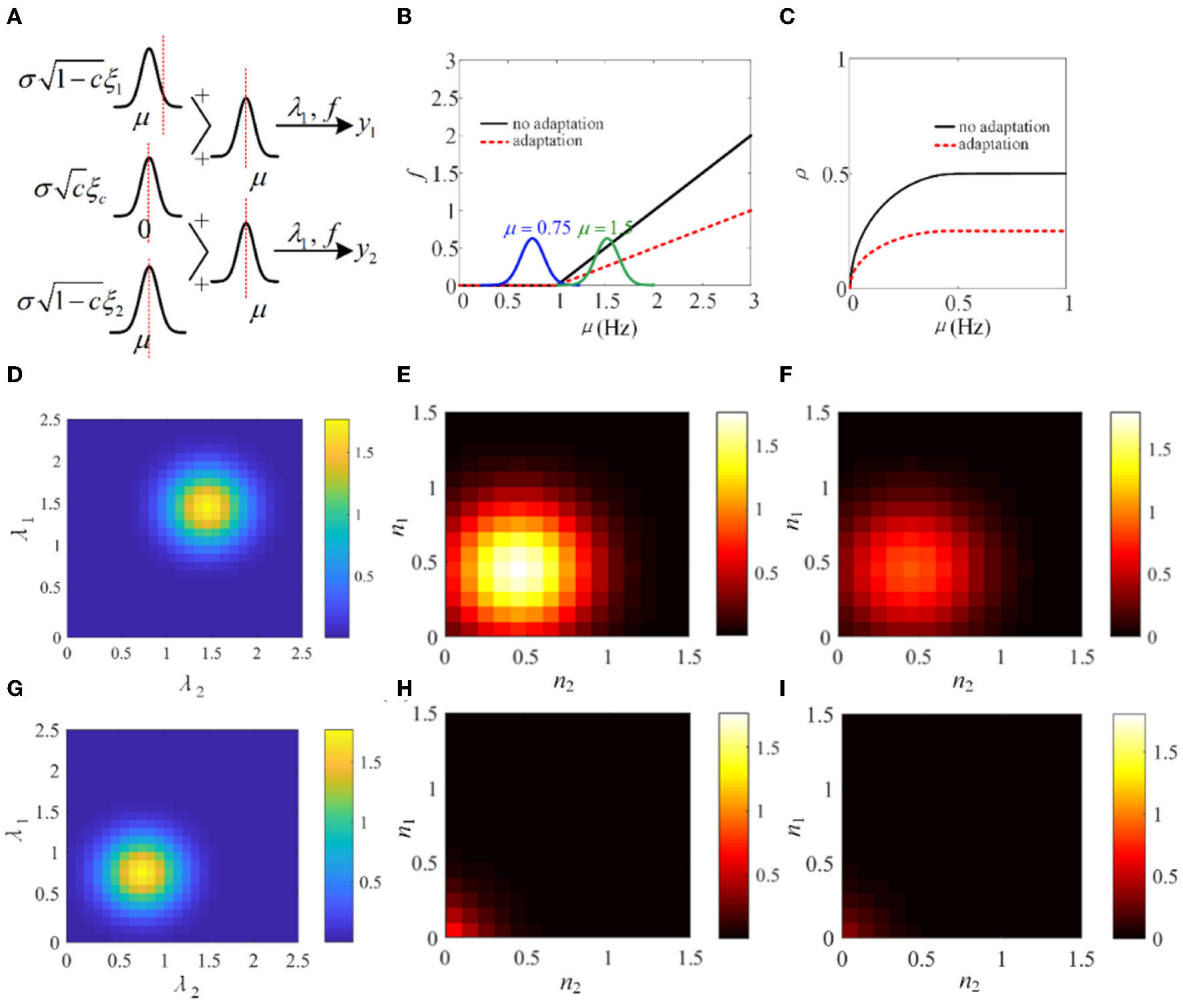
Adaptation mechanism analysis vs. correlation. **(A)** A phenomenological pairwise neuron model with input function *f*. λ_1_ and λ_2_ represent the input random variables that represent the summation of the shared and individual inputs to the two neurons. μ and σ are the parameters of the Gaussian distributions governing the statistics of the neural signals. This simplified model is analogy to the pairwise neurons in Figure 2. **(B)** The threshold-linear model. The function *f* has a higher slope without adaptation. **(C)** The output correlation variation with or without adaptation. **(D)** The input joint density *P*(λ_1_, λ_2_) under μ = 1.5. **(E)** The output density *P*(*y*_1_, *y*_2_) without adaptation. **(F)** The output joint density *P*(*y*_1_, *y*_2_) with adaptation under μ = 1.5. **(G)** The input joint density *P*(λ_1_, λ_2_) under μ = 0.75. **(H)** The input joint density *P*(λ_1_, λ_2_) with adaptation. **(I)** The output joint density *P*(*y*_1_, *y*_2_) with adaptation.

In Sections 3.1–3.4, the biological models and the phenomenological models are utilized to investigate the role of SFA in output correlation. The results show that SFA decreases the firing rate and further attenuates the output correlation of pairwise neurons. To make a mechanism analysis, a simple phenomenological model using a non-linear input function *f*, similar to that used in de la Rocha et al. (2007), is introduced. As shown in Figure 15A, λ_1_ and λ_2_ are in analogy to the pairwise neurons model. The input correlation is *c*, which is set to 0.5. The function *f* is set to threshold-linear. As shown in Figure 15B, the threshold-linear characteristic makes the model a rectifier. SFA decreases the slope of the linear portion of the rectifier. The output correlation is lower with adaptation compared with the output correlation without correlation (Figure 15C). The input distribution *P*(λ_1_, λ_2_) and the output distribution *P*(*y*_1_, *y*_2_) are depicted in Figures 15D, E. Furthermore, the output distribution with adaptation is plotted in Figure 15F. The introduction of adaptation decreases the correlation and response intensity. In addition, another Gaussian input, μ = 0.75 is investigated. The input distribution *P*(λ_1_, λ_2_) and the output distribution *P*(*y*_1_, *y*_2_) with or without adaptation are drawn in Figures 15G–I. The distribution is shifted to the lower parameter area. The eccentricity is smaller than that at μ = 1.5, which indicates that the external inputs play a pivotal role in the output correlation. The results show that when the Gaussian input overlaps with the non-linear area of function *f*, the output correlation is lower than the input correlation. As the eccentricity becomes lower for decreased input intensity, the output correlation attenuates. The eccentricity depends on the input correlation and the input intensity. In conclusion, SFA, through changing the input non-linearity of single neurons, may play an important role in the degree of correlation in a neural network.

## 4. Conclusion and discussion

We use conduction-based computation models applied with a dynamic threshold or adaptation current to analyze the mechanism of the SFA in modulating the output correlation. We demonstrate our results using several SFA mechanisms. We find that different SFA mechanisms modulate and decrease the output correlation by reducing the firing rate and the attenuated firing rate results in the correlation decrease. This correlation can be further decreased by enhanced adaptation conductance and decreased input intensity. In addition, different stimulation times divide the firing rate into two stages: rapid and sparse firing. These two processes caused the correlation to show an initial increase, followed by a steady reduction. The time and slide windows are found not to affect the decrease generated by the SFA. Furthermore, a simple phenomenological neuron model with a simple rectifier as a transfer function for the inputs supports the results. The mechanism indicates that the strength of the signal input and the slope of the linear component of the rectifier together determine the output correlation so that if SFA decreases the slope or the input signal decreases, output correlations will decrease.

The spike train output correlation depends on the input correlation and mean value of the correlated input. These factors suggest that the output correlation inherits the same trend as the mean firing rate variation *in vitro* cortical cells (de la Rocha et al., 2007; Doiron et al., 2016). Our results demonstrate these results by simulation experiments, and our simulation results matched well with the experimental results from the perspective of firing rate. In addition, inspired by the reduced modulation effect of the SFA on the firing rate (Ha and Cheong, 2017), we further prove that the SFA has a decreasing effect on the output correlation. The simulation results depend on the stimulation duration, and different stimulation durations have been shown to generate opposite results (Barreiro et al., 2012). Our study also found similar results: SFA presented opposite dynamic variations as stimulation time changed, resulting in opposite correlation variations. At the onset of stimulation, a transient adaptation current forms, generating a higher correlation coefficient when the ISIs are small. When the adaptation current became steady, the correlation remained lower, where the *ISIs* are offset to larger values. The correlation is calculated using the correlation coefficient related to time and slide windows. This correlation is proportional to the firing rate, and a larger time window generates a larger correlation (de la Rocha et al., 2007). Our results revealed a similar effect: a larger time window caused a stronger correlation in spike trains. However, the variation in the time window did not affect the decreasing tendency caused by the SFA on the correlation. This is because the variation in this factor only influences the correlation coefficient and does not affect the relationship between the SFA and correlation. Similar time and slide windows showed a less significant effect, consistent with a previous study where the windows are always fixed at a specific value (Shea-Brown et al., 2008; Litwin-Kumar et al., 2011). Nearby cortical neurons present a more synchronous response by enhancing the sensory drive strength (Kohn and Smith, 2005). This visual cortex experiment is consistent with the results in our study that the increased correlated inputs enhance the output correlation. The two neighboring ganglion cells generate a remarkably correlated response in the retina (Mastronarde, 1989). Our study investigates this correlated response from the biological perspective and further confirms that SFA is an important factor to decrease the correlation. In addition, the correlated response in the visual cortical area of monkeys is investigated in Bair et al. (2001); the results show that the cross-correlation is related to the short-term time scale, and the long-term scale is significant to the auto-correlation. This is consistent with the results that the time window plays a less important role in the long-time correlation between pairwise neurons.

This study reveals that SFA can decrease the output correlation between pairs of neurons with common inputs and can establish a connection between cellular mechanisms and information coding at the network level. However, the results of this study require further experimental validations *in vitro*. This study is a helpful supplement to the literature on a possible mechanism of population information coding from the perspective of SFA. It serves as a meaningful reference for further research on information coding strategies.

## Data availability statement

The original contributions presented in the study are included in the article/supplementary material, further inquiries can be directed to the corresponding author.

## Author contributions

JixW: ideas, development or design of methodology, and article writing. BD: formulation and evolution of overarching research goals and aims. TG: data analysis and theoretical derivation. JiaW: management and coordination responsibility for the research activity planning and execution. HT: development or design of methodology and creation of models. All authors contributed to the article and approved the submitted version.
